# Accurate and Affordable Vibrational Spectra of Large Molecules: Primary, Auxiliary, and Spectator Modes in a Perturb-then-Diagonalize Framework

**DOI:** 10.1021/acs.jctc.5c02123

**Published:** 2026-02-23

**Authors:** Vincenzo Barone, Federico Lazzari, Marco Mendolicchio

**Affiliations:** † 563196 INSTM, via G. Giusti 9, 50121 Firenze, Italy; ‡ 657489 Scuola Superiore Meridionale, Largo San Marcellino 10, 80138 Napoli, Italy; § 19004 Scuola Normale Superiore, Piazza dei Cavalieri 7, 56126 Pisa, Italy

## Abstract

Vibrational spectra convey a wealth of structural and dynamical information; however, their reliable assignment and interpretation often benefit from the integration of complementary spectroscopic techniques and require the support of accurate quantum chemical calculations. The harmonic approximation is frequently insufficient for quantitative spectroscopy, while fully anharmonic treatments rapidly become computationally prohibitive for large and flexible molecular systems, in particular, for biomolecules. In this framework, we introduce a general perturb-then-diagonalize approach that relies on a three-class partitioning of normal modes into primary, auxiliary, and spectator subsets and combines numerical strategies based on analytical Hessians and analytical gradients. Accurate anharmonic contributions are explicitly included for the modes of primary interest, while the influence of external modes is accounted for through finite differences of analytical gradients, avoiding the much more expensive evaluation of Hessians. Several case studies demonstrate the robustness, ease of use, and accuracy of the proposed approach across a broad range of molecular systems, including situations in which vibrational and rotational spectroscopic data provide complementary information. When combined with a dual-level strategy in which accurate methods are employed for harmonic terms and less expensive methods for anharmonic contributions, the present framework enables vibrational spectra of near-spectroscopic accuracy for biomolecules and other chemically rich systems. More complex environments can be addressed by coupling the method with multilayer approaches.

## Graphical abstract

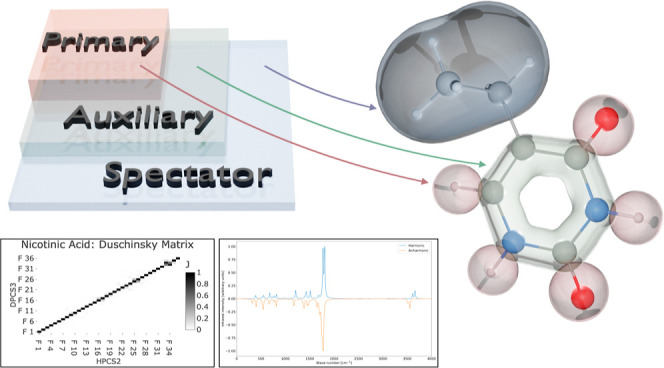

## 1 Introduction

Molecular spectroscopy is a technique of choice for probing the structural and dynamical properties of molecular systems in a noninvasive manner. In particular, recent advances in high-resolution spectroscopic techniques have made it possible to investigate gas-phase biomolecules and biorelevant molecular systems containing several tens of atoms. Gas-phase infrared (IR) spectra of such systems can now be recorded through rapid thermal heating of solid samples followed by fast-scan Fourier-transform (FT) IR spectroscopy prior to decomposition.^1,2^ Under the high-temperature conditions of these experiments, conformational cooling is suppressed, allowing access to a wide spectral window ranging from the near-to the mid-IR region for both IR and Raman spectroscopies.^3^

Quantum chemical methods therefore play a crucial role in the assignment of vibrational bands and in the interpretation of experimental results in terms of molecular structure and intra- and intermolecular interactions and are increasingly employed also by experiment-oriented scientists. However, describing biomolecules at a level of accuracy sufficient for reliable spectral assignments generally requires going beyond the standard rigid-rotor/harmonic-oscillator approximation, which is no longer adequate for a quantitative description of high-resolution spectra.^4^

A well-established route to improvement is the explicit inclusion of vibrational anharmonicity. Among the available approaches,^5,6^ vibrational perturbation theory, particularly in its second-order formulation (VPT2),^7,8^ has attracted considerable attention owing to its favorable balance between accuracy and computational efficiency compared to more demanding variational methods.^9–12^ In VPT2, anharmonic corrections are evaluated perturbatively starting from the harmonic reference, incorporating cubic and semidiagonal quartic force constants. In practice, these quantities are typically obtained by one-dimensional finite differences of analytical Hessians along the normal modes.^13^

In the present work, we build upon a general *perturb-then-diagonalize* strategy^14,15^ particularly suitable for reduced-dimensionality treatments.^16,17^ Within this framework, anharmonic corrections are first evaluated perturbatively (deperturbed VPT2, DVPT2), yielding effective vibrational energies in the absence of explicit resonance interactions. Subsequently, near-degenerate states are identified and treated through a variational diagonalization of an effective Hamiltonian (generalized VPT2, GVPT2), allowing the explicit inclusion of Fermi and Darling–Dennison resonances.^18^ More generally, this formulation permits the selective inclusion or exclusion of specific anharmonic contributions at the perturbative or variational stage of the overall protocol.

Despite their favorable cost-to-accuracy ratio, perturb-then-diagonalize approaches can still become computationally demanding for biomolecules, as the number of required force constant evaluations grows rapidly with system size. Moreover, in many experimental studies, only specific portions of the vibrational spectrum are of primary interest and are rich in diagnostically relevant information. These considerations have motivated the development of reduced-dimensionality strategies in which anharmonic corrections are computed explicitly only for selected subsets of normal modes associated with the spectral regions of interest. While such approaches substantially reduce the computational cost, they often neglect important anharmonic couplings with the remaining modes, potentially leading to inaccurate results.

Within this context, it is worth noting that contributions of decreasing magnitude but intrinsically increasing computational cost can be most effectively treated using electronic-structure methods of progressively lower cost rather than concentrating the entire computational effort on a single aspect of the calculation. This perspective, which is at the heart of the Pisa Composite Schemes philosophy,^19,20^ has already proved highly successful for the accurate determination of equilibrium molecular structures and ground-state rotational constants, enabling a reliable interpretation of high-resolution rotational spectra for medium-sized and biorelevant molecules.^21,22^ The present developments naturally extend this hierarchical strategy to vibrational spectroscopy, providing a unified framework in which structural information derived from rotational spectroscopy and anharmonic vibrational effects can be treated in a consistent and mutually reinforcing manner.

Building on these considerations, the present work introduces an improved reduced-dimensionality framework based on a three-class partitioning of the vibrational normal modes into primary, auxiliary, and spectator subsets. This classification is fully consistent with the perturb-then-diagonalize strategy: auxiliary modes play the role of perturbers, governing the anharmonic coupling network, while primary modes correspond to the spectroscopic observables of interest and can be treated either perturbatively or variationally depending on the presence of resonances. The remaining spectator modes are retained at the harmonic level. In this scheme, analytical Hessians are computed for displacements along the primary modes of interest but only analytical gradients along the auxiliary modes; the resulting hierarchical treatment captures the dominant anharmonic effects accurately while retaining a balanced description of mode coupling at a fraction of the cost of full-dimensional calculations.

Representative case studies were selected to assess the accuracy, robustness, and flexibility of the proposed framework across different regimes of molecular complexity. A small semirigid molecule (HFCO) is first employed as a benchmark system to validate the internal consistency of the reduced-dimensionality strategy.

Nitrobenzene and uracil are then considered as examples of rigid aromatic molecules containing non innocent substituents, allowing the assessment of selective mode treatment in the presence of localized anharmonic effects and, possibly, vibrational resonances.

Larger conjugated molecules (indene and pyrene) are investigated to probe the performance of the method in the presence of delocalized vibrational manifolds and dense spectral patterns.

A biologically relevant molecule, such as glycine, is subsequently used to illustrate the challenges posed by strong mode coupling and flexibility in biomolecular spectroscopy.

Finally, nicotinic acid is included as a representative case in which the integration of complementary spectroscopic techniques—namely, rotational and vibrational spectroscopy—provides stringent and mutually reinforcing structural information, enabling an unambiguous conformational assignment.

The paper is organized as follows. The next section summarizes the theoretical background and computational details of the proposed approach. Subsequently, results obtained with different partitions into primary, auxiliary, and spectator modes are compared for the selected case studies. The performance and practical applicability of the method are then discussed. Finally, the conclusions and perspectives are presented.

## 2 Theoretical Background

The transition energy from a vibrational state *S*, characterized by quantum numbers {*v*_S,*i*_}, to a state R, characterized by quantum numbers {*v*_R,*i*_}, can be written as

1 $$\nu_{RS}=\sum_{i=1}^{N}\Delta v_{RS,i}\omega_{i}+\sum_{i=1}^{N}\sum_{j=i}^{N}\chi_{ij}\left[\Delta_{2}v_{RS,i}+\frac{1}{2}\left(\Delta v_{RS,i}+\Delta v_{RS,j}\right)\right]$$

where ω_*i*_ is the *i*th wavenumber (in cm^–1^), Δ*v*_RS,*i*_ = *v*_R,*i*_ – *v*_S,*i*_, and Δ_2_ *v*_RS,*ij*_ = *v*_R,*i*_ *v*_R,*j*_ – *v*_S,*i*_ *v*_S,*j*_. The diagonal and off-diagonal elements of the **χ** matrix are given by

2 $$\chi_{ii}=\frac{1}{16}Y_{ii}-\frac{1}{32}\sum_{\begin{array}{c} j=1\\ \left(j\neq i\right) \end{array}}^{N}Z_{ij}$$

3 $$\chi_{ij}=\frac{1}{4}Y_{ij}-\frac{1}{8}\sum_{\begin{array}{c} k=1\\ \left(k\neq i,j\right) \end{array}}^{N}Z_{ijk}$$

The **Y** matrix accounts for the direct anharmonic contribution of the considered mode or pair of modes:

4 $$Y_{ii}=\eta_{iiii}-\frac{\sigma_{iii}^{2}/2+9\rho_{iii}^{2}/2}{3\omega_{i}}$$

5 $$Y_{ij}=\eta_{iijj}-\frac{1}{2}\left[\frac{\sigma_{iij}^{2}}{2\omega_{i}+\omega_{j}}+\frac{\rho_{iij}^{2}}{2\omega_{i}-\omega_{j}}\right]-\frac{1}{2}\left[\frac{\sigma_{jji}^{2}}{2\omega_{j}+\omega_{i}}+\frac{\rho_{jji}^{2}}{2\omega_{j}-\omega_{i}}\right]-\frac{\rho_{iii}\rho_{ijj}}{\omega_{i}}-\frac{\rho_{jjj}\rho_{jii}}{\omega_{j}}$$

whereas the **Z** tensor accounts for the indirect effect of modes other than those explicitly appearing in the *Y* matrix:

6 $$Z_{ij}=\frac{4\rho_{jii}^{2}}{\omega_{j}}+\frac{\sigma_{iij}^{2}}{2\omega_{i}+\omega_{j}}-\frac{\rho_{iij}^{2}}{2\omega_{i}-\omega_{j}}$$

7 $$Z_{ijk}=\frac{\sigma_{ijk}^{2}}{\omega_{i}+\omega_{j}+\omega_{k}}-\frac{\rho_{ijk}^{2}}{\omega_{i}+\omega_{j}-\omega_{k}}+\frac{\rho_{ikj}^{2}}{\omega_{i}-\omega_{j}+\omega_{k}}-\frac{\rho_{jki}^{2}}{\omega_{i}-\omega_{j}-\omega_{k}}+\frac{2\rho_{kii}\rho_{kjj}}{\omega_{k}}$$

In the above expressions,

8 $$\eta_{ijkl}=f_{ijkl}+g_{ij,kl}+g_{kl,ij}$$

9 $$\sigma_{ijk}=f_{ijk}-\left(g_{ij,k}+g_{ik,j}+g_{jk,i}\right)$$

10 $$\rho_{ijk}=f_{ijk}-\left(g_{ij,k}-g_{ik,j}-g_{jk,i}\right)$$

where *f* and *g* denote derivatives of the potential and kinetic energy, respectively, with respect to normal coordinates. The well-known expressions for Cartesian coordinates^11^ are recovered by setting η_*ijkl*_ = *f*_*ijkl*_ and σ_*ijk*_ = ρ_*ijk*_ = *f*_*ijk*_ and by adding to *Y*_*ij*_ the Coriolis contribution

11 $$C_{ij}=\frac{4\left(\omega_{i}^{2}+\omega_{j}^{2}\right)}{\omega_{i}\omega_{j}}\sum_{\tau =x,y,z}B_{\tau }^{eq}{\left\{\zeta_{ij,\tau }\right\}}^{2}$$

where ζ_*ij*,τ_ is the Coriolis coupling constant and *B*_τ_^eq^ is the corresponding equilibrium rotational constant.

The required cubic and semidiagonal quartic force constants can be obtained from finite differences of analytical force constant (Hessian) matrices expressed in dimensionless normal coordinates.^13^ Within this framework, the computation of Hessians at geometries displaced in both directions along the different normal modes provides access to all of the terms required to evaluate transition energies involving the primary modes, with the exception of some contributions arising when the index *j* corresponds to an auxiliary mode.

A well-known alternative strategy is based on the use of analytical gradients computed at the same displaced geometries to perform the finite-difference procedure:^23,24^

12 $$f_{iij}=\frac{f_{j}\left(+\delta q_{i}\right)+f_{j}\left(-\delta q_{i}\right)-2f_{j}\left(q_{eq}\right)}{\delta q_{i}^{2}}$$

13 $$f_{iiii}=3\frac{f_{i}\left(+\delta q_{i}\right)-f_{i}\left(-\delta q_{i}\right)-2\omega_{i}\delta q_{i}}{\delta q_{i}^{3}}$$

In this case, displacements along single normal modes provide access to all the required force constants except for three-index cubic terms and semidiagonal quartic force constants. Although approaches based on energy differences instead of gradients have also been proposed,^25,26^ the number of required sampling points increases dramatically, from 1 + 2*N* to 1 + 4*N* + 2*N*(*N* – 1). This increase is not compensated by any practical advantage, since the computational cost of gradients is generally lower than or at most comparable to that of single-point energy evaluations for most quantum chemical methods.

An alternative and more efficient strategy exploits the fact that the definition of normal modes requires a preliminary Hessian calculation, which nearly doubles the computational effort of the pure gradient-based approach. In this context, an approximate Hessian can be computed at a low level (LL) of theory, and the corresponding normal modes can be used to define the displacements for the numerical differentiation of high-level (HL) analytical gradients, employing step sizes appropriate for the evaluation of quadratic force constants. The approximate cubic force constants obtained at this stage can then be used to identify modes associated with small anharmonic contributions, which can therefore be confidently treated at the harmonic level and classified as spectator modes.

Subsequently, the approximate Hessian is diagonalized, and the resulting high-level normal modes are employed to generate displacements for the nonspectator modes, using step sizes optimized for the evaluation of anharmonic force constants. In this latter step, increased numerical accuracy can be achieved by employing four-point finite-difference stencils instead of the standard two-point schemes:

14 $$f_{iij}=\frac{1}{12\delta q_{i}^{2}}\left[16f_{j}\left(+\delta q_{i}\right)+16f_{j}\left(-\delta q_{i}\right)-30f_{j}\left(q_{eq}\right)-f_{j}\left(+2\delta q_{i}\right)-f_{j}\left(-2\delta q_{i}\right)\right]$$

15 $$f_{iiii}=\frac{1}{2\delta q_{i}^{3}}\left[f_{i}\left(+2\delta q_{i}\right)-f_{i}\left(-2\delta q_{i}\right)-2f_{i}\left(+\delta q_{i}\right)+2f_{i}\left(-\delta q_{i}\right)\right]$$

Since the evaluation of three-index cubic and semidiagonal quartic force constants from analytical gradients requires displacements along pairs of normal modes, the use of analytical Hessians becomes more convenient whenever these contributions are needed. On this regard, nonspectator modes are further divided into primary and auxiliary modes. For primary modes, anharmonic contributions are obtained from finite differences of analytical Hessians, whereas for auxiliary modes, they are computed from finite differences of analytical gradients. In both cases, only one-dimensional displacements along the individual normal modes are employed.

With this strategy, the diagonal elements of the block of the χ matrix corresponding to both the primary and auxiliary modes are exact. The same holds for off-diagonal elements coupling primary modes among themselves, as well as for those coupling primary and auxiliary modes.

On the other hand, the *Y*_*ij*_ elements coupling primary (*i*) and spectator (*j*) modes lack the last term of eq 5, namely, ρ_*iij*_ρ_*jjj*_/(4ω_*j*_), while those coupling auxiliary and spectator modes lack this contribution as well as the η_*iijj*_ term. As a consequence, the latter term is also missing in the coupling between auxiliary modes.

All *Z* terms involving only spectator modes are not available, whereas the *Z*_*ij*_ terms are exact whenever at least one of the involved modes belongs to the auxiliary or primary subsets. Finally, for the *Z*_*ijk*_ terms, all contributions except 2ρ_*kii*_ρ_*kjj*_/ω_*k*_ cannot be included when none of the three modes belong to the primary subset.

Despite these approximations, the proposed three-class strategy is considerably more accurate and flexible than the conventional two-mode force-field approximation. In particular, the present approach allows selected three-index cubic force constants to be retained, which is essential for the proper description of Fermi resonances. These resonances can induce non-negligible frequency shifts, as explicitly shown by the structure of eq 7.

It is also worth noting that, as recently demonstrated,^27,28^ vibrational corrections to molecular properties, including rotational constants, do not require the explicit inclusion of primary modes. In fact, auxiliary modes are sufficient to determine all of the relevant anharmonic contributions. As a result, the determination of auxiliary modes at a high level (HL) of theory—at a computational cost comparable to that of a harmonic frequency calculation—allows one to fully account for vibrational averaging effects while simultaneously capturing the leading anharmonic contributions to vibrational frequencies.

This observation is consistent with the fundamental assumption underlying low-order perturbative approaches, namely, that anharmonic corrections are small (typically below 5%) with respect to the zero-order harmonic terms. Consequently, cubic and quartic force constants require a lower level of accuracy in comparison to their quadratic counterparts. At the same time, harmonic frequencies are generally more sensitive to the quality of the underlying quantum chemical model than higher-order force constants^29^ while being significantly less demanding from a computational point of view.

These considerations motivated the development of dual-level^12^ (or hybrid^4^) approaches. In the simplest additive scheme,^30^ VPT2 results obtained at a low level (LL) of theory are corrected using the differences between LL harmonic frequencies and their counterparts computed at a high level (HL). A more robust strategy, known as the substitution model,^11^ employs HL harmonic frequencies together with LL anharmonic contributions in the solution of the VPT2 equations. In the hybrid gradient approach described above, LL semidiagonal cubic and diagonal quartic force constants are obtained as a byproduct of the harmonic frequency calculation and can therefore be combined with LL harmonic frequencies to solve the VPT2 equations.

While the substitution model generally provides reliable results, it requires that LL and HL normal modes be very similar and that possible inversions in the ordering of the modes be properly addressed. This limitation is overcome by the recently proposed all-in approach, in which HL normal modes are employed for the finite-difference evaluation of LL cubic and semidiagonal quartic force constants. All three approaches are implemented in the general VPT2 engine employed in the present study.

Finally, when curvilinear internal coordinates are adopted, the required derivatives of the kinetic energy can be obtained by finite differences concurrently with those of the potential energy.^15,28^

A quantitative criterion for the classification of vibrational modes into primary, auxiliary, and spectator subsets can be formulated by noting that the dominant neglected contributions contain a known factor, α_τ_ = ρ_*ii*τ_/ω_τ_, with τ = *j* or *k*. The absolute value |α_τ_| can therefore be employed as a screening parameter for the different normal modes.^16,17^ In the present framework, auxiliary modes are sufficient to perform this screening, so analytical Hessians need to be computed only once, after the primary modes have been selected on the basis of auxiliary-mode information alone. Furthermore, when the hybrid gradient approach is employed, displacements along LL normal modes provide approximate HL estimates of all the *f*_τττ_, *f*_ρρτ_, and ω_τ_ quantities required for a more accurate screening procedure.

Alternatively, after the selection of primary modes (for instance, by imposing a frequency window), the similarity between a primary mode *P* and a spectator mode *S* can be quantified through

16 $$\beta_{PS}=\sum_{i=1}^{N_{c}}\left|L_{iP}\right|\left|L_{iS}\right|$$

where *N*_c_ is the number of coordinates used to construct the normal modes. Spectator modes for which β_PS_ exceeds a predefined threshold for at least one primary mode are reclassified as auxiliary modes.

The intensities of vibrational transitions depend on the derivatives of appropriate molecular properties, such as the dipole moment for infrared spectroscopy or the polarizability for Raman spectroscopy. In this context, energy gradients yield the underlying property values (Ω), and their numerical differentiation provides access to the harmonic (Ω_*i*_) and leading anharmonic (Ω_*ii*_) contributions:

17 $$\Omega_{i}={\left(\frac{\partial \Omega }{\partial q_{i}}\right)}_{eq}=\frac{\Omega \left(+\delta q_{i}\right)-\Omega \left(-\delta q_{i}\right)}{\delta q_{i}}$$

18 $$\Omega_{ii}={\left(\frac{\partial^{2}\Omega }{\partial q_{i}^{2}}\right)}_{eq}=\frac{\Omega \left(+\delta q_{i}\right)+\Omega \left(-\delta q_{i}\right)-2\Omega \left(q^{eq}\right)}{\delta q_{i}^{2}}$$

The quantity Ω_*i*_ is directly available from the analytical Hessian evaluated at the equilibrium geometry. These contributions are therefore accessible for auxiliary modes, whereas semidiagonal second- and third-order derivatives (Ω_*ij*_ and Ω_*iij*_) are additionally available for primary modes through numerical differentiation of property gradients computed concurrently with analytical Hessians.

In this connection, overtones and combination bands are of particular relevance since their intensities vanish within the harmonic approximation. The VPT2 expression for the transition moment associated with these bands can be written as

19 $${\left\langle \Omega \right\rangle }_{0,\left(1+\delta_{ij}\right)\left(1+\delta_{ij}\right)}=\sqrt{\frac{2}{1+\delta_{ij}}}[\frac{s_{1}S}{2}\left(\Omega_{ij}+\Omega_{ji}\right)+\frac{s_{0}}{4}\sum_{k=1}^{N}f_{ijk}\Omega_{k}\left(\frac{S}{\omega_{i}+\omega_{j}-\omega_{k}}-\frac{1}{\omega_{i}+\omega_{j}+\omega_{k}}\right)]$$

where *S*, *s*_0_, and *s*_1_ are constants whose values depend on the specific molecular property and are reported in ref 31 for the most common spectroscopic techniques. All of the terms entering eq 19 are available whenever at least one of the indices *i* or *j* corresponds to a primary mode. Moreover, the transition moment of the first overtones, obtained by setting *i* = *j* in eq 19, is fully determined even when *i* is an auxiliary mode.

The situation is more involved for fundamental transitions. Based on the complete expressions reported in refs 17 and 31, all required terms are available when *i* is a primary mode and all other modes are auxiliary, whereas some contributions involving spectator modes are missing. As a consequence, exact results can be obtained for selected spectral windows through a reduced-cost strategy requiring 2*N*_P_ Hessian evaluations and 2­(*N* – *N*_P_ – *N*_S_) gradient evaluations, where *N* is the total number of normal modes and *N*_P_ and *N*_S_ are the numbers of primary and spectator modes, respectively. For large molecular systems, modes belonging to distant spatial regions or spectral ranges can be safely treated at the harmonic level with negligible loss of accuracy and a substantial additional reduction of computational cost.

The different reduced-dimensionality models introduced in this section will be assessed in the following sections by means of representative case studies and different quantum chemical descriptions. Before the results are presented, however, the next section outlines the computational details including the choice of quantum chemical models and the dual-level treatment of harmonic and anharmonic contributions.

## 3 Computational Details

Throughout this work, the notation QM′//QM″ indicates a dual-level vibrational treatment in which harmonic frequencies and normal modes are computed at the QM′ level, whereas anharmonic corrections are evaluated at the QM″ level. This separation is particularly advantageous for anharmonic vibrational calculations, where different contributions to the force field exhibit markedly different sensitivities to the level of electronic-structure theory.

In most cases, LL computations were carried out at the B3LYP/6-31+G* level, including Grimme’s D3BJ empirical dispersion corrections.^32^ The corresponding HL computations were performed using the revDSD-PBEP86-D3BJ functional^33^ in conjunction with a slightly modified cc-pVTZ-F12 basis set^34^ (hereafter denoted as 3F12^–^ ^21^), in which d functions are omitted on hydrogen atoms and the two f functions on heavier atoms are replaced by a single f function taken from the cc-pVTZ basis set.^35^ Following the systematic nomenclature of the so-called Pisa composite schemes,^36^ these two levels are referred to as HPCS2 and DPCS3, respectively.^36^

In selected cases, molecular structures and harmonic contributions were further refined at the PCS2 level, which is based on explicitly correlated coupled-cluster theory.^36,37^

All DFT calculations were performed using the Gaussian program package^38^ and the coupled-clusters ones by the Molpro package^39^ via an in-house interface.^40,41^ In-house scripts were then employed to follow the general three-class reduced-dimensionality workflow described in this work.

## 4 Results and Discussion

Normal modes expressed in Cartesian coordinates were employed for all of the systems investigated in this work. This choice is motivated by the fact that anharmonic mechanical and electrical contributions to transition moments are not yet available for curvilinear coordinates, thereby preventing a consistent treatment of intensities within the present framework.

The set of molecular systems investigated in this work has been selected to probe the performance of the proposed reduced-dimensionality framework across different regimes of structural complexity and vibrational behavior, with a particular emphasis on biorelevant molecules. The test cases include both relatively rigid and semirigid systems, as well as more flexible molecules characterized by low-frequency modes and pronounced anharmonic couplings. In addition, conjugated and mixed conjugated–nonconjugated frameworks are considered in order to assess the ability of the method to describe dense vibrational manifolds and delocalized vibrational patterns.

Special attention is devoted to systems for which the integration of complementary spectroscopic techniques provides stringent and mutually reinforcing structural information. In this context, nicotinic acid is included as a representative example of a molecule whose conformational behavior can be unambiguously characterized only by combining highly accurate rotational constants with selectively refined anharmonic vibrational spectra. This case study illustrates the ability of the present framework to support a coherent interpretation of rotational and vibrational data within a unified hierarchical computational strategy.

### 4.1 Fluoroformaldehyde

The first test case is fluoroformaldehyde (Figure 1), a small semirigid molecule that nonetheless exhibits non-negligible anharmonic effects. Owing to its limited size and well-characterized vibrational spectrum, HFCO represents a convenient benchmark system for assessing the performance and internal consistency of reduced-dimensionality strategies before addressing more complex, biorelevant molecules.

**Figure 1:**
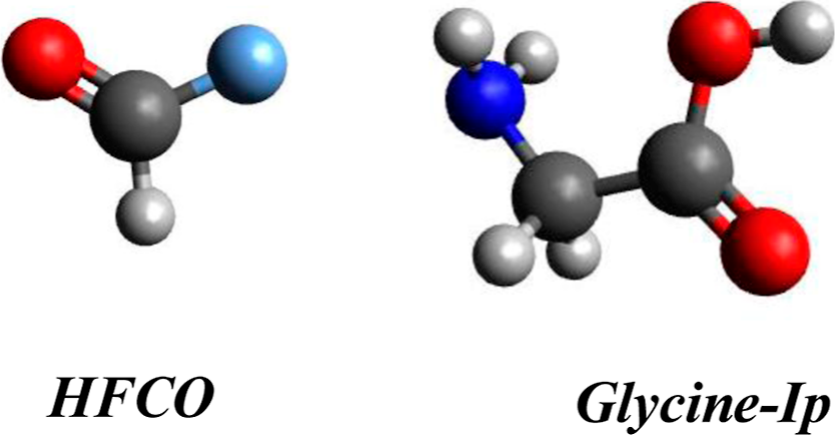
Molecular structures of fluoroformaldehyde and glycine-Ip.

The results reported in Table 1 clearly highlight the importance of anharmonic corrections, particularly for stretching modes, and demonstrate the ability of the dual-level DPCS3//HPCS2 approach to closely reproduce full DPCS3 results at a significantly reduced computational cost. As expected for this system, the DVPT2 and GVPT2 predictions are nearly identical, reflecting the absence of relevant Fermi or Darling–Dennison resonances.

**Table 1:** Harmonic and Anharmonic Fundamental Wave Numbers of Fluoroformaldehyde (in cm^–1^) at Different Levels of Theory

		HPCS2^a^			DPCS3^b^			DPCS3//HPCS2^c^		DPCS3//HPCS2^d^		
assign.	symm.	harm	DVPT2	GVPT2	harm	DVPT2	GVPT2	DVPT2	GVPT2	DVPT2	GVPT2	exp.^e^
CH str.	A′	3134	2988	2982	3125	2991	2985	2991	2985	2990	2984	2981
CO str	A′	1894	1861	1861	1867	1834	1834	1836	1836	1834	1834	1837
HCO bend	A′	1372	1340	1340	1377	1345	1345	1348	1348	1346	1346	1342
CF str	A′	1058	1033	1033	1080	1053	1053	1054	1054	1054	1054	1065
FCO bend	A′	650	643	643	666	658	658	659	660	659	659	663
oop bend	A″	1020	1003	1003	1035	1018	1018	1019	1019	1017	1017	1011

a Anharmonic treatment at the HPCS2 level of theory.

b Anharmonic treatment at the DPCS3 level of theory.

c Anharmonic treatment obtained by combining gradient and Hessian calculations at the DPCS3 level for the C–H stretching mode, and from DPCS3 gradients and HPCS2 Hessians for all other modes.

d Anharmonic treatment obtained by combining gradient and Hessian calculations at the DPCS3 level for the C=O stretching mode, and from DPCS3 gradients and HPCS2 Hessians for all other modes.

e From ref 42.

Particularly noteworthy are the results obtained with the new three-class strategy, in which DPCS3 analytical Hessians are computed only along selected primary modes (either the C=O or the C–H stretchings), while analytical gradients are evaluated for the remaining auxiliary modes. The missing semidiagonal quartic force constants are recovered at the lower HPCS2 level. This hierarchical treatment yields anharmonic fundamental frequencies that are virtually indistinguishable from the full DPCS3 reference values.

From a computational perspective, this strategy is especially appealing because the cost of a single DPCS3 Hessian evaluation is comparable to that of the full set of 6*N* – 11 HPCS2 Hessians required to build a complete cubic and semidiagonal quartic force field. As a result, most of the accuracy of a high-level anharmonic treatment can be retained at a fraction of the computational cost.

### 4.2 Substituted Aromatic Systems: Nitrobenzene and Uracil

As discussed in Theoretical Background, the dominant contributions neglected in conventional reduced-dimensionality models are associated with terms of the form

20 $$R_{ij}=\frac{f_{iii}f_{ijj}}{\omega_{i}}$$

which account for part of the coupling between primary and spectator modes but are fully taken into account in the couplings between primary and auxiliary modes.

Nitrobenzene (Figure 2) provides a particularly instructive test case in this context. On the one hand, it is a relatively rigid aromatic molecule, and in the other, it contains a nitro group that is known to be poorly described at low levels of theory. Nitrobenzene therefore constitutes an ideal system for assessing both the robustness of the proposed reduced-dimensionality framework and its ability to selectively identify and accurately treat problematic vibrational modes.

**Figure 2:**
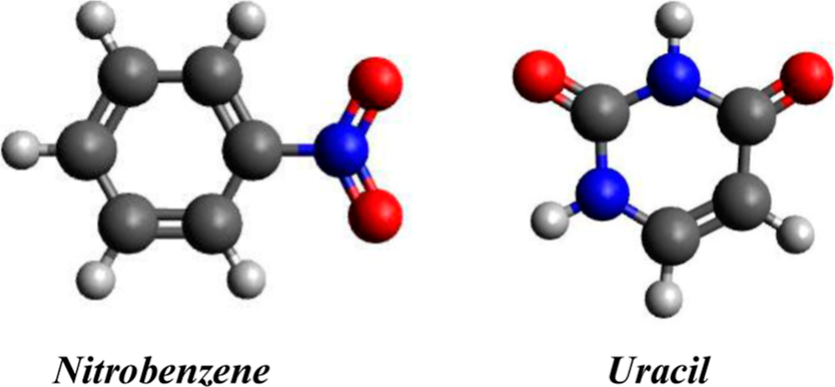
Molecular structure of nitrobenzene and uracil.

The behavior of the *R*_*ij*_ terms is analyzed in detail for nitrobenzene by inspecting the distribution of the absolute values of the elements of the **R** matrix. As shown in the histogram reported in Figure 3, significant contributions are confined to a limited number of modes, whereas the vast majority of the vibrational space is characterized by rapidly decaying |*R*_*ij*_| values. This finding strongly suggests that for most modes, the neglected contributions are expected to have only a minor impact on the resulting anharmonic frequencies.

**Figure 3:**
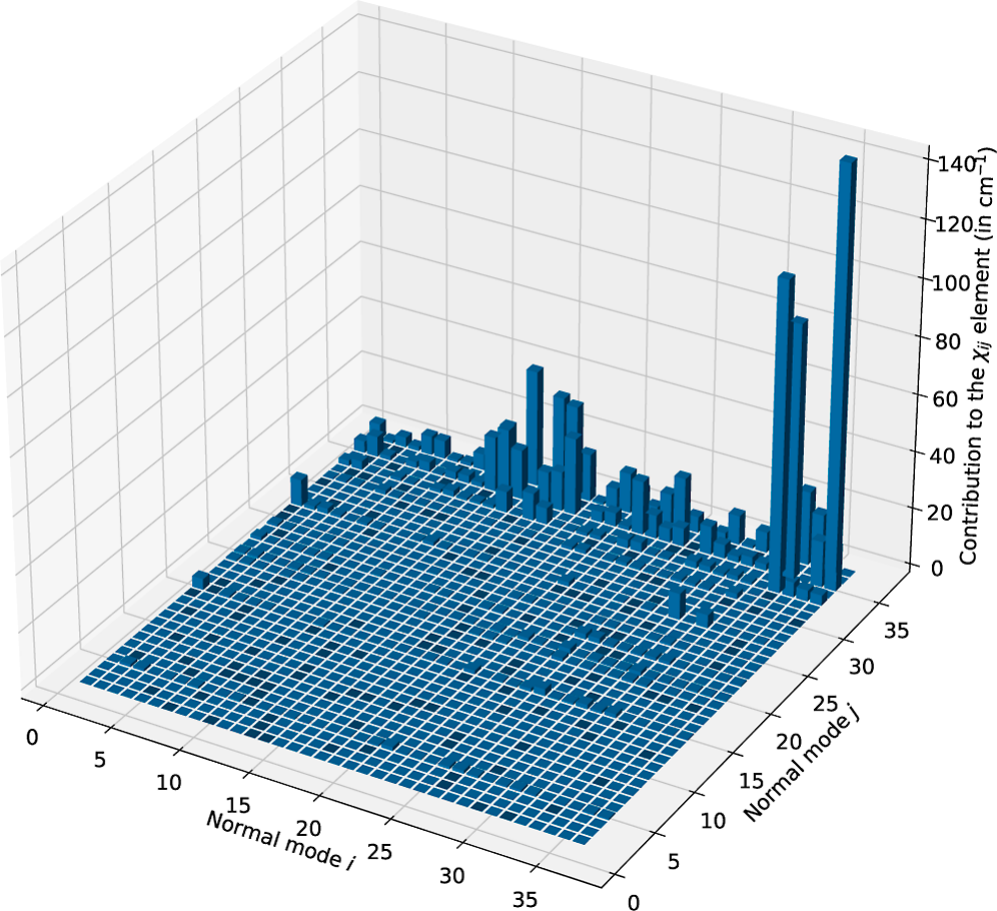
Absolute values of the elements of the **R** matrix of nitrobenzene at the HPCS2 level of theory. All values are in cm^–1^.

This expectation is confirmed by the values of the C–H stretching fundamentals summarized in Table 2. Even when only five modes are considered primary, standard reduced-dimensionality (RD) calculations already provide a satisfactory description of the anharmonic spectrum. The inclusion of augmented reduced-dimensionality corrections (RD-aug), which selectively account for the dominant neglected couplings, further improves the agreement with the full-dimensional reference, bringing the RD-aug prediction into near-perfect alignment with the full calculation.

**Table 2:** Analysis of the Anharmonic Fundamental Wavenumbers (in cm^–1^) of Nitrobenzene C–H Stretching Modes at the HPCS2 Level of Theory

assignment	symmetry	harm	full	RD	RD-aug
sym. str.	A_1_	3255	3115	3118	3115
sym. str.		3219	3092	3095	3092
sym. str.		3198	3090	3093	3090
asym. str.	B_2_	3255	3118	3121	3118
asym. str.		3210	3087	3090	3087

Uracil (Figure 2) represents a paradigmatic example of a biomolecular system in which strong anharmonic couplings and vibrational resonances play a central role in shaping the infrared spectrum.^43–45^ In particular, the two C=O stretching modes are involved in pronounced Fermi and Darling–Dennison interactions with lower-frequency modes, making uracil a stringent test for perturbative vibrational approaches.

As illustrated in Figure 4, anharmonic corrections induce substantial changes in both peak positions and relative intensities, with respect to the harmonic spectrum. In the present reduced-dimensionality treatment, the anharmonic spectrum is obtained by evaluating analytical gradients at the lower level of theory while displacing the system along high-level normal modes and by computing analytical Hessians at the higher level for all stretching vibrations, which are treated as primary modes.

**Figure 4:**
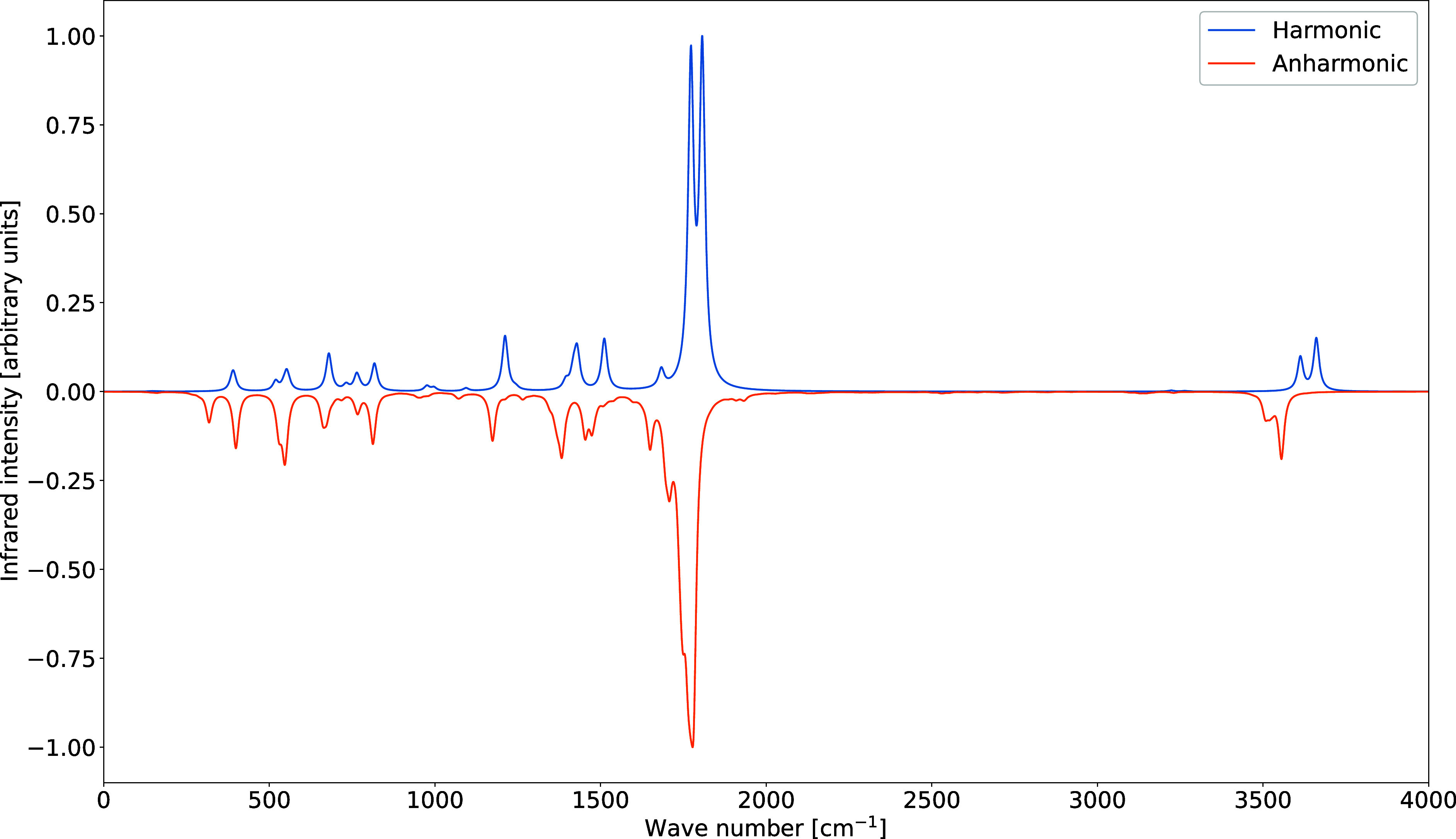
Comparison between the harmonic and anharmonic theoretical IR spectra of uracil. Spectral line shapes have been convoluted by Lorentzian distribution functions with HWHMs of 10 cm^–1^.

The quantitative data reported in Table 3 confirm that anharmonic effects are particularly pronounced for N–H, C–H, and C=O stretching vibrations, with shifts exceeding 150 cm^–1^ in several cases. The close agreement with experimental reference values demonstrates that an accurate description of these strongly anharmonic modes can be achieved within a reduced-dimensionality framework, provided that chemically relevant vibrations are treated at an appropriately high level and that resonance effects are properly accounted for through the perturb-then-diagonalize procedure.

**Table 3:** Harmonic and Anharmonic Wave Numbers (in cm^–1^) Associated with the Stretching Modes of Uracil

		harm		anharm		
assignment	symmetry	DPCS3	PCS2	DPCS3	PCS2//DPCS3	exp.^a^
N1–H1 str.	A′	3661	3653	3496	3488	3484
N2–H2 str.	A′	3613	3602	3448	3437	3436
C–H3 str.	A′	3262	3253	3136	3127	3124
C–H4 str.	A′	3222	3217	3088	3083	3084
C=O1 str.	A′	1807	1790	1777	1760	1764
C=O2 str.	A′	1773	1762	1746	1735	1728

a From refs 44 and 45 .

### 4.3 Large Aromatic Systems: Indene and Pyrene

Among the molecular systems considered in this work, indene and pyrene (Figure 5) provide representative examples of conjugated aromatic hydrocarbons of increasing size and vibrational complexity. While indene can be regarded as a moderately sized aromatic molecule featuring both sp^2^- and sp^3^-hybridized carbon atoms, pyrene represents a prototypical polycyclic aromatic hydrocarbon and constitutes a relevant reference for larger systems of interest in materials science and astrochemistry.

**Figure 5:**
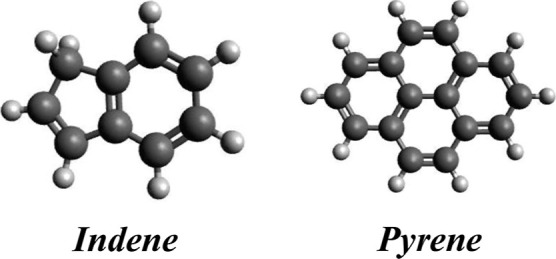
Molecular structures of indene and pyrene.

For indene, anharmonic effects play a dominant role in shaping the vibrational spectrum in the X–H stretching region. This observation naturally suggests a reduced-dimensionality treatment in which analytical Hessians at the higher level (DPCS3) are computed exclusively for the C–H stretching modes, which are therefore classified as primary, while all remaining anharmonic contributions are evaluated at the lower level (HPCS2). Sizeable anharmonic red shifts are observed for all C–H stretching fundamentals, with mode-dependent displacements spanning approximately 130–170 cm^–1^. These shifts are not uniform across the stretching manifold, reflecting the different local chemical environments associated with aromatic sp^2^ C–H bonds and the sp^3^-hybridized methylene group, as well as their distinct anharmonic coupling patterns.

The resulting anharmonic IR spectrum is in remarkable overall agreement with its gas-phase experimental counterpart^46^ (Figure 6), confirming that a selective high-level treatment of the stretching modes is sufficient to capture the dominant anharmonic features governing the experimental profile.

**Figure 6:**
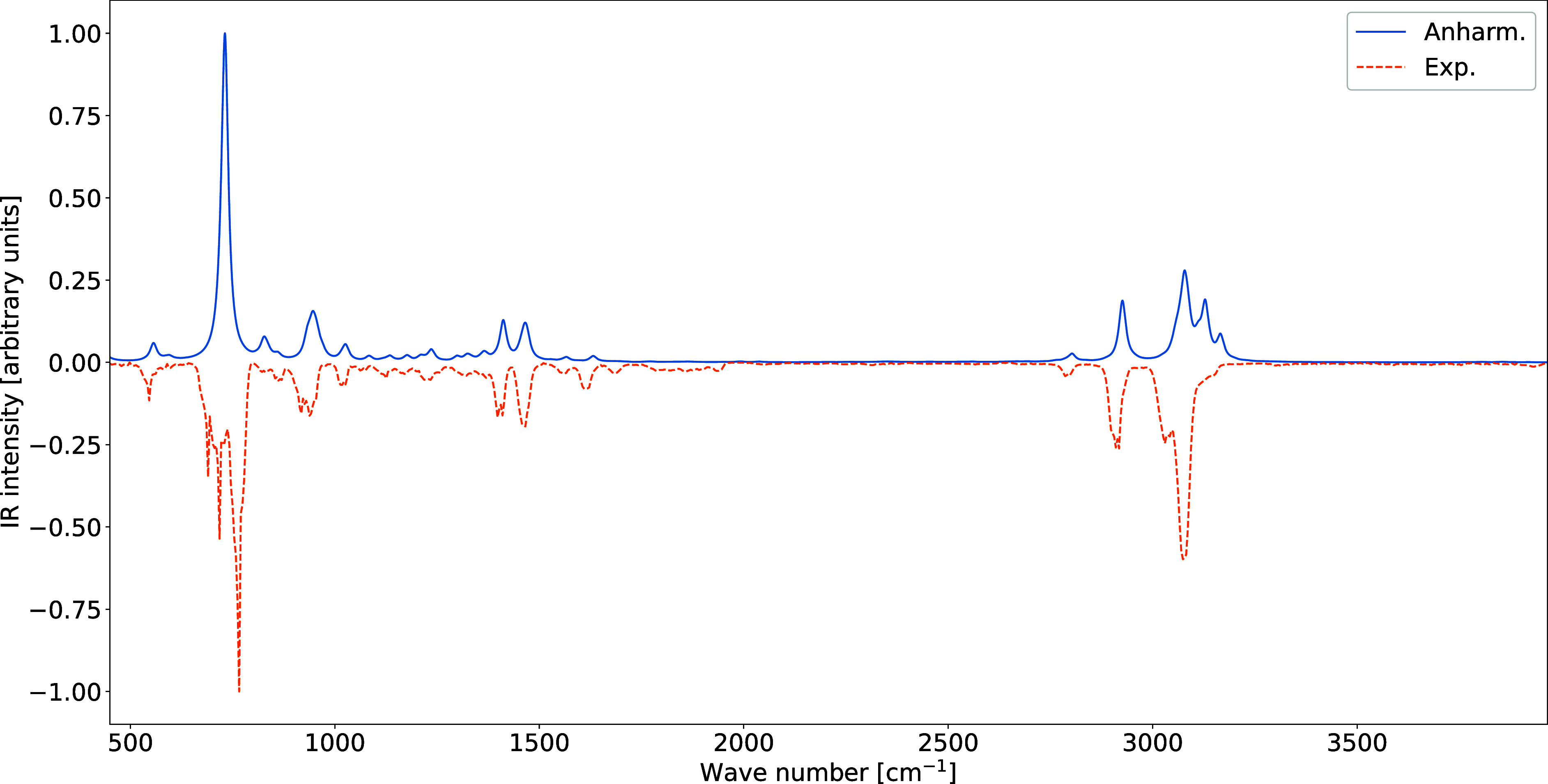
Comparison between the theoretical (anharmonic) and experimental IR spectra of indene. Spectral line shapes have been convoluted by Lorentzian distribution functions with HWHMs of 10 cm^–1^.

An analogous reduced-dimensionality computation was performed for pyrene, which contains 27 atoms and is characterized by a dense vibrational manifold and strongly delocalized normal modes. Despite the increased molecular size and the absence of localized sp^3^ sites, the selective treatment of the C–H stretching modes as primary again proves sufficient to recover an IR spectrum in very good agreement with the gas-phase experiment (Figure 7).

**Figure 7:**
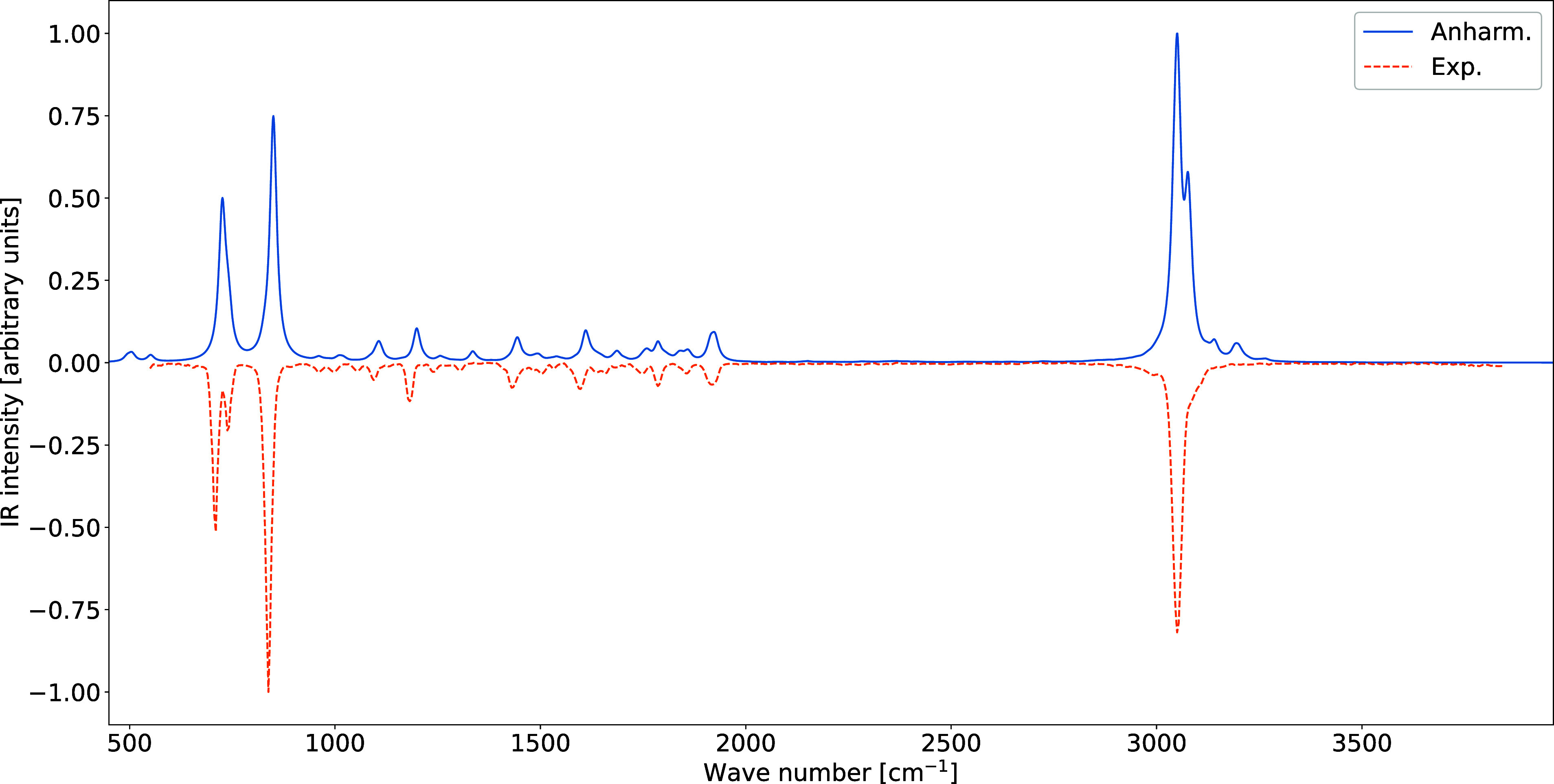
Comparison between the theoretical (anharmonic) and experimental IR spectra of pyrene. Spectral line shapes have been convoluted by Lorentzian distribution functions with HWHMs of 10 cm^–1^.

Overall, the indene and pyrene case studies demonstrate that the proposed three-class reduced-dimensionality strategy remains robust and transferable for extended aromatic systems, even in the presence of strong mode delocalization and a high density of vibrational states.

### 4.4 Flexible Systems: Glycine (Ip Conformer)

A more demanding test is provided by flexible molecules for which low-frequency motions and extended mode couplings can significantly affect the vibrational spectrum. As a representative biomolecular example, we consider the Ip conformer of glycine (Figure 1), which combines a relatively small size with pronounced anharmonic effects arising from internal flexibility and X–H stretching vibrations.

Table 4 compares harmonic and anharmonic fundamental frequencies obtained with different computational models against available experimental data.^3,47–49^ In the reduced-dimensionality substitution (RD-Sub) approach labeled as Set I, harmonic frequencies and normal modes are computed at the DPCS3 level, while anharmonic corrections are evaluated by using HPCS2 calculations displaced along the DPCS3 normal coordinates. In Set II, the same protocol is adopted, but analytical Hessians along the X–H stretching modes are computed directly at the DPCS3 level, thereby selectively increasing the accuracy for chemically critical vibrations.

**Table 4:** Comparison between Theoretical and Experimental Fundamental Wavenumbers (cm^–1^) of the Ip Conformer of Glycine^a^

		DPCS3		DPCS3//HPCS2		
assignment	symmetry	harm.	anharm.	set I^b^	set II^c^	exp.
OH str.	A′	3771	3580	3581	3580	3585
NH_2_ s str.		3523	3383	3368	3371	3359
CH_2_ s str.		3067	2953	2937	2941	2943
C=O str.		1817	1785	1786	1789	1779
NH_2_ bend		1685	1632	1602	1632	1608
CH_2_ bend		1474	1438	1438	1442	1429
CH_2_ bend		1418	1393	1379	1395	1405
(OH + CH_2_) bend		1318	1299	1255	1303	1297
CN str. + OH bend		1177	1140	1139	1138	1136
C=O str. + OH bend		1139	1105	1105	1106	1101
CC str. + NH_2_ bend		937	887	880	887	883
CC str.		835	811	811	805	801
(NH_2_ + OCO) bend		639	639	635	634	636
CCO(H) bend		468	462	462	461	464
CCN bend		260	262	262	261	250
NH_2_ as str.	A″	3600	3431	3418	3428	3410
CH_2_ as str.		3109	2962	2950	2963	2969
CH_2_ bend		1397	1360	1359	1362	1340
CH_2_ NH_2_ twist		1195	1168	1168	1169	1166
CH_2_ NH_2_ twist		922	911	914	917	907
OH oop bend		651	624	638	607	615
OH oop bend		512	503	498	502	500
CN tors. (ϕ)		215	212	206	238	204
CC tors. (ψ)		68	101	83	120	
MAE (cm^–1^)		58.3	8.9	9.8	9.4	
MAX (cm^–1^)		190	24	42	34	

a Modes are ordered by decreasing experimental frequency, with A′ modes listed first and A″ modes second. Mean absolute errors (MAEs) and maximum absolute errors (MAXs) are computed with respect to experimental values

b Anharmonic calculation performed through the RD-Sub method (higher level: DPCS3, lower level: HPCS2).

c Same protocol as in footnote *a*, with the X–H stretching modes treated at the DPCS3 level (see text for details).

The results clearly show that selectively computing high-level Hessians for the X–H stretching modes leads to a systematic improvement in the predicted anharmonic frequencies, particularly in the high-frequency region. This trend is reflected in the reduced deviations from the experiment, demonstrating that selectively increasing the level of theory for a small subset of chemically relevant modes can yield substantial gains in accuracy without incurring the cost of a fully high-level anharmonic treatment.

### 4.5 Nicotinic Acid: Integration of Rotational and Vibrational Spectroscopy

The combined use of different spectroscopic techniques often provides a much more stringent and informative characterization of the molecular structure than any single experiment alone. In particular, rotational spectroscopy delivers highly accurate structural fingerprints through ground-state rotational constants, whereas vibrational spectroscopy probes local chemical environments and anharmonic effects. A consistent theoretical framework capable of interpreting both types of data at comparable levels of accuracy is, therefore, highly desirable.

In this context, the hierarchical strategies developed within the Pisa Composite Schemes (PCS) framework^19,36^ have already demonstrated their reliability for the prediction of rotational constants of medium-sized molecules, enabling unbiased conformational assignments through direct comparison with microwave spectra.^20^ Extending this level of accuracy to vibrational spectroscopy while retaining computational affordability represents a natural and important step toward a unified spectroscopic description.

As a representative example of this integrated approach, we consider nicotinic acid, also known as vitamin B3. Nicotinic acid plays a central role in cellular metabolism as a precursor of the various forms of the coenzyme nicotinamide adenine dinucleotide (NAD)^50^ and is also widely employed in pharmacology as a cholesterol-lowering agent.^51^ As for many biologically relevant molecules, their chemical activity is closely related to their molecular structure and conformational behavior.

Nicotinic acid exhibits two nearly isoenergetic planar conformers, commonly termed s-cis and s-trans, which differ by the relative orientation of the carboxylic group with respect to the pyridine ring (see Figure 8).

**Figure 8:**
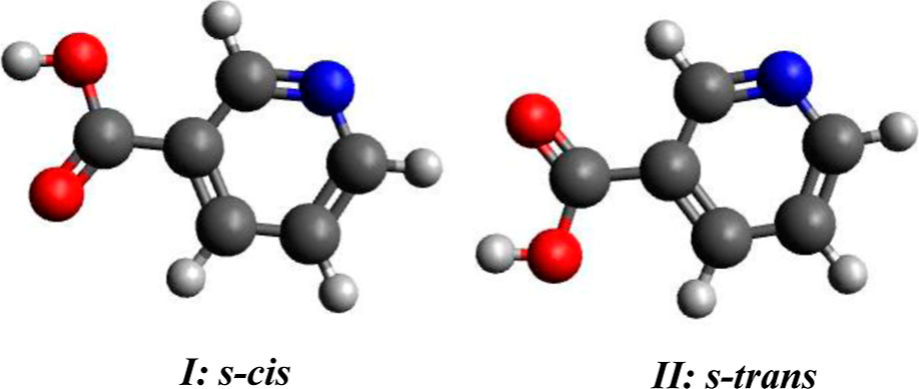
Low-energy conformers of nicotinic acid.

Owing to their close structural similarity, the infrared spectra of the two conformers are nearly indistinguishable, whereas their rotational spectra provide clear fingerprints that allow an unambiguous conformational discrimination.^52^

The results reported in Table 5 show that ground-state rotational constants computed by including vibrational corrections are in significantly better agreement with the experiment than the corresponding equilibrium values. In particular, the purely equilibrium rotational constants obtained at the DPCS3 and PCS2 levels display deviations that are too large to permit a reliable conformational assignment, clearly demonstrating that equilibrium structures alone are not sufficient for quantitative rotational spectroscopy of nicotinic acid.

**Table 5:** Ground-State Rotational Constants (MHz) of Nicotinic Acid for the Two Lowest-Energy Conformers^a^

	rotational constants			errors Δ		
method	*A*	*B*	*C*	Δ*A*	Δ*B*	Δ*C*
Conformer I						
DPCS3	3959.3	1247.3	948.5	+15.1	+2.2	+1.5
PCS2	3968.9	1252.1	951.8	+24.7	+7.0	+4.8
PCS2//HPCS2	3941.0	1244.3	946.3	–3.2	–0.8	–0.7
BDPCS3//HPCS2	3945.2	1243.5	946.0	+1.0	–1.6	–1.0
exp.	3944.2	1245.1	947.0			
Conformer II (Δ*E* = 93.7 cm^–1^, Δ*H*_0_ = 91.2 cm^–1^)						
DPCS3	3962.7	1245.8	947.8	+14.7	+2.1	+1.4
PCS2	3972.5	1250.7	951.2	+24.5	+7.0	+4.8
PCS2//HPCS2	3944.8	1242.9	945.6	–3.2	–0.8	–0.8
BDPCS3//HPCS2	3948.9	1242.0	945.3	+0.9	–1.7	–1.1
exp.	3948.0	1243.7	946.4			

a Experimental values from ref 52 are truncated to one decimal place for consistency with the reported errors, although their actual precision is significantly higher. Errors (Δ) are defined as Calc. – Exp.

When vibrational corrections are included, both PCS2//HPCS2 and BDPCS3//HPCS2 yield rotational constants that are in very good agreement with the experimental data for both conformers, enabling a straightforward and unbiased discrimination between the s-cis and s-trans forms. It is worth noting that differences between computed and experimental rotational constants below approximately 0.1% are not physically meaningful in this context, since the overall accuracy is then dominated by the uncertainty associated with the vibrational corrections evaluated at the HPCS2 level. Importantly, within the present framework, these vibrational corrections are obtained as a direct byproduct of the anharmonic force-field evaluation for the auxiliary and, when required, primary modes, and therefore do not entail any additional computational overhead.

Among the investigated models, PCS2 represents the reference approach, as it is based on a coupled-cluster explicitly correlated treatment including core–valence effects and does not rely on any empirical parameters. As expected, PCS2 consistently outperforms the DPCS3 equilibrium description. Remarkably, however, the BDPCS3 model—employing a single empirical parameter together with a priori bond-length corrections—achieves an accuracy that is competitive with that of PCS2 once vibrational effects are taken into account while retaining a computational cost comparable to that of standard DFT methods.

A preliminary insight into the vibrational description of nicotinic acid can be obtained by comparing the normal modes computed at different electronic-structure levels. To this end, the overlap (Duschinsky) matrix between the normal modes obtained at the HPCS2 and DPCS3 levels is reported in Figure 9.

**Figure 9:**
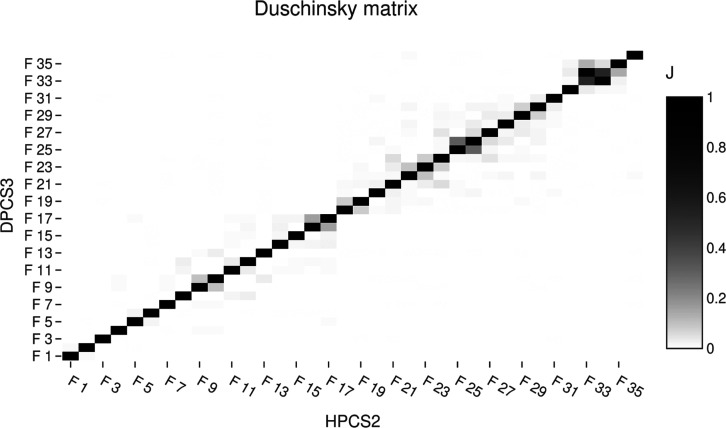
Duschinsky matrix between HPCS2 and DPCS3 normal modes of nicotinic acid (conformer I).

The close correspondence between the two sets of normal modes is immediately apparent, indicating that the two descriptions provide a largely consistent harmonic picture of the vibrational manifold. As a consequence, the overall harmonic IR spectra computed at the two levels are very similar, with the notable exception of the N–H stretching band around 3700 cm^–1^, whose frequency is underestimated by about 75 cm^–1^ at the HPCS2 level.

The GVPT2 IR spectrum obtained treating all modes at the HPCS2 level with the exception of a DPCS3 description of the N–H stretching mode is in remarkable agreement with its gas-phase experimental counterpart^46^ (see Figure 10) while maintaining an affordable computational cost.

**Figure 10:**
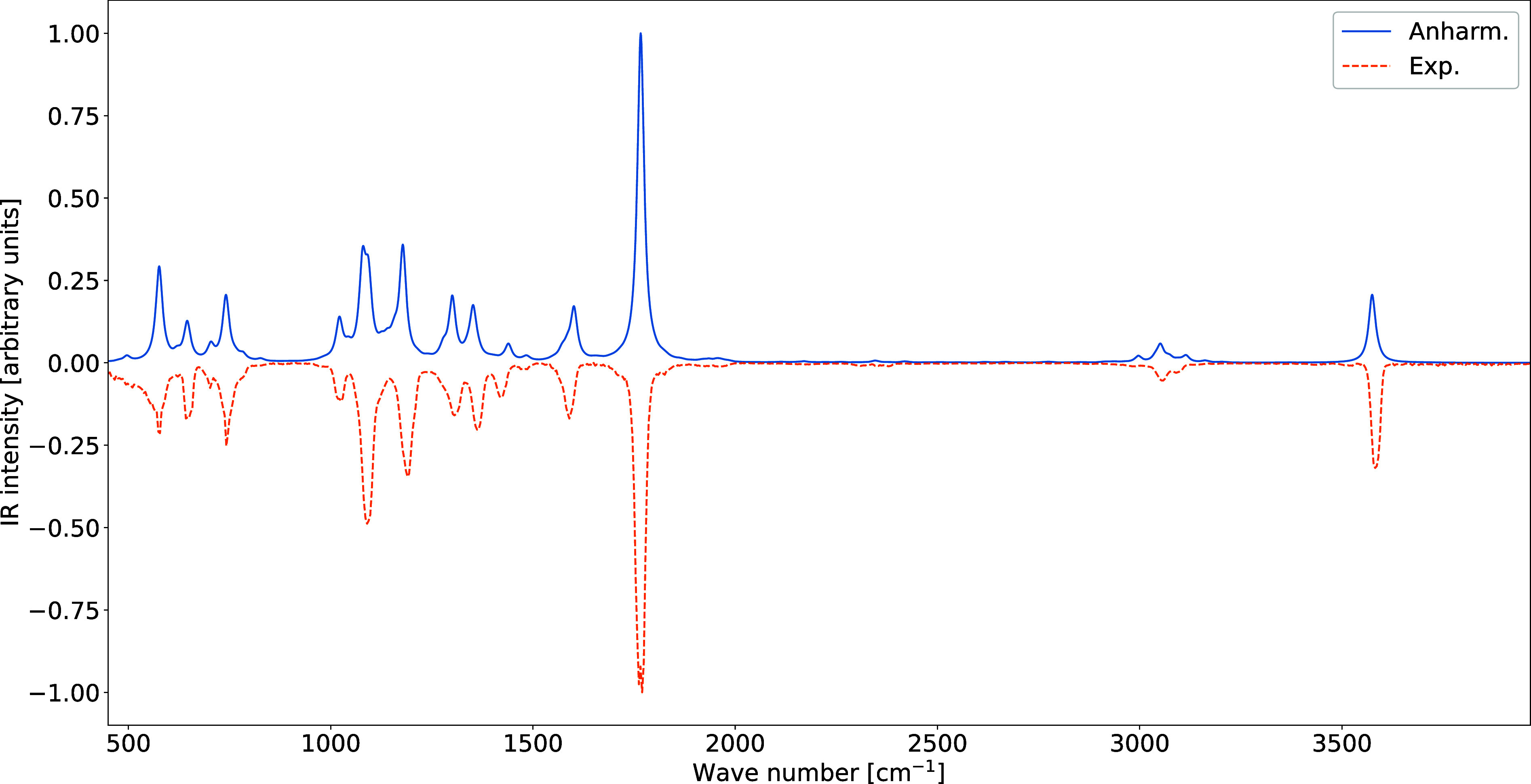
Dual-level GVPT2 (dashed) and gas-phase experimental (solid) IR spectrum of nicotinic acid (conformer I). Spectral line shapes have been convoluted by Lorentzian distribution functions with HWHMs of 10 cm^–1^.

Overall, this analysis demonstrates how the PCS/GVPT2 strategy, combined with a selective dual-level treatment of anharmonic effects, enables a coherent and quantitatively reliable interpretation of vibrational spectra. Together with the results obtained from rotational spectroscopy, this approach provides a unified and hierarchical framework for the spectroscopic characterization of complex molecular systems.

### 4.6 General Assessment of the Proposed Framework

The set of case studies presented above provides a coherent picture of the strengths and limitations of the proposed three-class reduced-dimensionality framework across molecular systems of increasing size, flexibility, and anharmonic complexity. Despite the chemical diversity of the investigated molecules, several common trends clearly emerge from the analysis, including cases in which complementary spectroscopic techniques probe different but interrelated aspects of the molecular structure.

First, the results consistently show that anharmonic effects play a crucial role not only for high-frequency X–H stretching modes but also for medium- and low-frequency vibrations whenever non-negligible mode couplings are involved. In all systems considered, purely harmonic treatments fail to reproduce experimental trends, whereas even partially anharmonic descriptions lead to substantial improvements. This behavior is particularly evident when vibrational spectroscopy is combined with highly accurate rotational data, which provide stringent structural constraints that cannot be reconciled with harmonic vibrational models alone.

Second, the comparison between full and reduced-dimensionality calculations demonstrates that the dominant anharmonic contributions are often governed by a relatively small subset of vibrational modes. When these modes are treated explicitly at a higher level, the remaining vibrational degrees of freedom primarily act as a perturbing environment. This behavior is clearly reflected in the analysis of semidiagonal coupling terms, which are found to be significant only for a limited number of mode pairs, even in medium-sized aromatic and heterocyclic systems. As a consequence, carefully designed reduced-dimensionality models can capture the essential physics of anharmonic vibrational spectra without resorting to a complete treatment.

Third, the results highlight the importance of flexibility in defining the reduced vibrational space. Rigid molecules with weak resonances, flexible biomolecular motifs, and systems characterized by multiple nearly isoenergetic conformers benefit from different choices of primary and auxiliary modes. The present framework accommodates these differences naturally, allowing the vibrational treatment to be tailored to the specific spectroscopic problem under investigation. In this respect, the use of chemically intuitive criteria—such as mode localization, resonance analysis, spectral window selection, or consistency with rotationally derived structural assignments—proves to be an effective and robust practical strategy.

Finally, the numerical evidence confirms that combining reduced-dimensionality vibrational treatments with hierarchical and dual-level electronic-structure descriptions represents a viable compromise between accuracy and computational cost. In all examined cases, the selective use of higher-level treatments for chemically or spectroscopically critical modes leads to systematic improvements while avoiding the steep scaling associated with uniformly high-level anharmonic force fields. This balance is particularly important when vibrational and rotational spectroscopic data are interpreted within a unified framework as it allows both types of observables to be described with comparable accuracy.

Overall, the present results support the view that reduced-dimensionality anharmonic treatments, when formulated within a flexible and physically motivated framework, can provide reliable and internally consistent spectroscopic predictions well beyond the reach of conventional harmonic approaches, paving the way for routine applications to increasingly complex molecular systems and multispectroscopic analyses.

## 5 Conclusions

A general reduced-dimensionality vibrational framework has been proposed and implemented, combining a three-class partitioning of vibrational modes with a dual-level electronic-structure strategy. The present results demonstrate that this approach enables an accurate and computationally affordable evaluation of selected spectroscopic features, extending near-spectroscopic accuracy to molecular systems that are beyond the reach of fully anharmonic treatments.

The case studies analyzed in detail provide practical guidelines for the definition of reduced vibrational spaces tailored to specific spectroscopic targets. In particular, normal modes falling within predefined spectral windows and/or localized in chemically relevant regions (such as X–H stretching vibrations) can be treated explicitly at a higher level, while the remaining modes can be included at a lower level or retained at the harmonic approximation. The analysis of semidiagonal coupling terms further supports the identification of a limited subset of auxiliary modes that mediate the dominant anharmonic interactions.

Compared to conventional two-class reduced-dimensionality models, the present framework retains the influence of auxiliary modes on the primary ones through one-dimensional numerical differentiation of analytical gradients, avoiding the much higher cost associated with Hessian-based treatments. When combined with a dual-level description of harmonic and anharmonic contributions, this strategy provides a balanced compromise between accuracy and computational efficiency, allowing chemically relevant vibrational effects to be described without resorting to uniformly high-level anharmonic force fields.

An additional strength of the proposed approach lies in its ability to support the consistent interpretation of complementary spectroscopic techniques. As exemplified by the combined analysis of rotational and vibrational spectra, highly accurate rotational constants—reliably predicted within the established PCS hierarchy—provide stringent structural constraints that can be naturally integrated with selectively refined anharmonic vibrational treatments. This synergy enables an unambiguous conformational assignment and a coherent structural interpretation that cannot be achieved by either technique alone.

The flexibility of the proposed framework makes it suitable for a wide range of molecular systems, from relatively rigid species to flexible biomolecular motifs and conjugated aromatic frameworks. Moreover, its formulation naturally allows for extensions toward multilayer descriptions, enabling the treatment of environmental effects and complex chemical contexts within the same conceptual framework. While further developments will be required to address large-amplitude motions, metal-containing systems, and open-shell species, the present results already demonstrate that reduced-dimensionality anharmonic treatments, when combined with physically motivated mode selection, hierarchical electronic-structure models, and multispectroscopic integration, provide a reliable and practical route for the structural interpretation of vibrational spectra in complex molecular systems.
